# Supplementary material for: Temperature and feeding frequency impact the survival, growth, and metamorphosis success of Solea solea larvae
Source: PLoS One. 2023 Mar 23;18(3):e0281193. doi: 10.1371/journal.pone.0281193 (PMC10035848; doi:10.1371/journal.pone.0281193)
Supplement: S1 File — (DOCX) [file pone.0281193.s001.docx]

**Supplementary information for**

**Temperature and feeding frequency impact the survival, growth, and metamorphosis success of *Solea solea* larvae**

Adriana E. Sardi^a^, Marie-Laure Bégout^b^, Anne-Laure Lalles^a^, Xavier Cousin^b^, Hélène Budzinski^a^

a University of Bordeaux, CNRS, EPOC, UMR 5805, F-33400, Talence, France

b MARBEC, Univ Montpellier, CNRS, Ifremer, IRD, F-34250 Palavas-Les-Flots, France

*** Corresponding author:**
Dr. Adriana E. Sardi ORCID: https://orcid.org/0000-0001-5323-2006
adrianasardi@gmail.com

Table S1. Chronology of the experimental conditions for rearing *S. solea* larvae in 24-well microplates and number of *Artemia salina* provided to larvae during the whole pilot experiment.

| **age (dph)** | **Length measured** | **Water changed** | **Artemia/well** | **Comments** |
| --- | --- | --- | --- | --- |
| 1 |  |  |  | Hatching |
| 2 |  |  |  |  |
| 3 |  | x |  | Transfer to microplates |
| 4 |  |  |  |  |
| 5 |  | x | 5 | Start feeding |
| 6 |  |  | 5 |  |
| 7 | x | x | 7 |  |
| 8 |  |  | 14 | Artemia added for Saturday and Sunday |
| 9 |  |  |  | Not fed |
| 10 |  | x | 15 |  |
| 11 |  |  | 20 |  |
| 12 |  | x | 20 |  |
| 13 |  |  | 20 |  |
| 14 | x | x | 20 |  |
| 15 |  |  | 20 |  |
| 16 |  |  | 20 |  |
| 17 |  | x | 20 |  |
| 18 |  |  | 25 |  |
| 19 |  | x | 25 |  |
| 20 |  |  | 30 | Start of metamorphosis |
| 21 | x | x | 30 |  |
| 22 |  |  | 70 | Artemia added for Saturday and Sunday |
| 23 |  |  |  | Not fed |
| 24 |  | x | 40 |  |
| 25 |  |  | 40 |  |
| 26 |  | x | 40 |  |
| 27 |  |  | 40 |  |
| 28 |  | x | 50 |  |
| 29 |  |  | 80 | Artemia added for Saturday and Sunday |
| 30 |  |  |  | Not fed |
| 31 |  | x | 50 |  |
| 32 |  |  | 50 |  |
| 33 |  |  |  | End of the experiment |

Table S2. Food densities in Experiment 1 (# of Artemias per individual). The amount of food was calculated based on results from our pilot experiment. Due to high mortality, the experiment only lasted until larvae were 16 days post-hatching.

| **Age (dph)** | **High food (100%)** | **Moderate food (60%)** | **Low food (40%)** |
| --- | --- | --- | --- |
| 5 | 5 | 4 | 2 |
| 6 | 5 | 4 | 2 |
| 7 | 10 | 7 | 3 |
| 8 | 10 | 7 | 3 |
| 10 | 15 | 11 | 5 |
| 11 | 15 | 11 | 5 |
| 12 | 20 | 14 | 6 |
| 13 | 20 | 14 | 6 |
| 14 | 20 | 14 | 6 |
| 15 | 20 | 14 | 6 |
| 16 | 20 | 14 | 6 |
| 17 | 20 | 14 | 6 |
| 18 | 20 | 14 | 6 |
| 19 | 30 | 21 | 9 |
| 20 | 30 | 21 | 9 |
| 21 | 35 | 25 | 11 |
| 22 | 35 | 25 | 11 |
| 23 | 40 | 28 | 12 |
| 24 | 40 | 28 | 12 |
| 25 | 40 | 28 | 12 |
| 26 | 40 | 28 | 12 |
| 27 | 40 | 28 | 12 |
| 28 | 50 | 35 | 15 |
| 29 | 50 | 35 | 15 |
| 30 | 50 | 35 | 15 |
| 31 | 50 | 35 | 15 |
| 32 | 50 | 35 | 15 |
| 33 | 50 | 35 | 15 |

Table S3. Analysis of variance using permutation test (PERMANOVA) for the total length (until 35 dph) and dry weight (until 22 dph) from larvae reared at different temperatures and feeding regimes. Statistically significant differences are highlighted in bold. Abbreviations stand for F: pseudo-F-ratio; R2: coefficient of determination, P: probability of *F*.

| Source of variation dry weight data | *F* | R2 | *P* |
| --- | --- | --- | --- |
| Temperature (T) | 0.015 | 0.0002 | 0.89 |
| Feeding frequency (F) | 0.611 | 0.0171 | 0.53 |
| T x F | 0.515 | 0.0144 | 0.59 |

| Source of variation length data | *F* | R2 | *P* |
| --- | --- | --- | --- |
| Temperature (T) | 0.0001 | 0 | 0.99 |
| Feeding frequency (F) | 7.4943 | 0.135 | **< 0.001** |
| T x F | 1.53 | 0.027 | 0.082 |

Figure S1. Kaplan-Meir curves comparing survival probability of feeding regimes treatments at the two experimental temperatures. Mantel-Cox test or log-rank test is a is non-parametric and hypothesis-based for comparing the distribution of survival curves of at least two samples. The test is convenient when the data are asymmetric or censored (in our case, the censored data correspond to the individuals taken for biometrics).


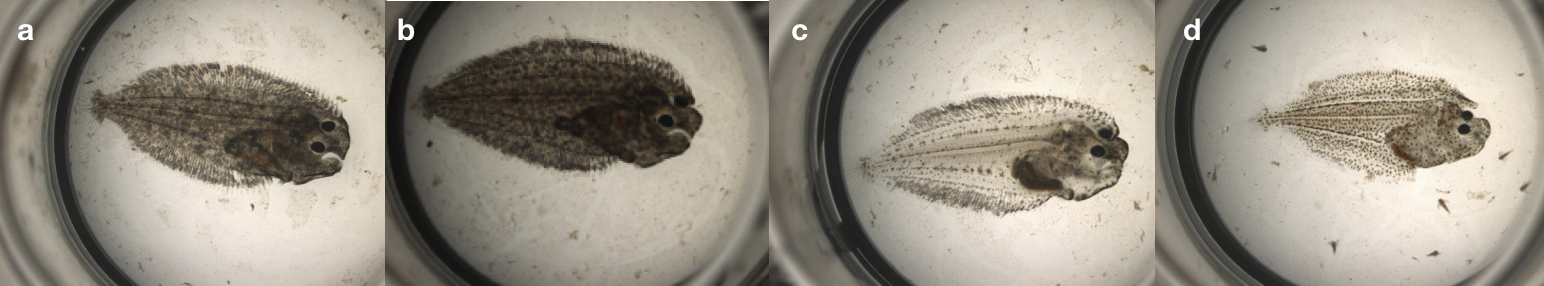


Figure S2. Examples of metamorphosed larvae and observed malformations or errors in regular metamorphosis, a) fully and normal metamorphosed larvae (stage 4), b) larva with an incomplete eye migration and incomplete head reshaping, c) larva with an incomplete eye migration and completed head reshaping (head is not oval), and d) larva with a hook-shaped piece of flesh above the head. All larvae were 35 dph and reared at 20°C.


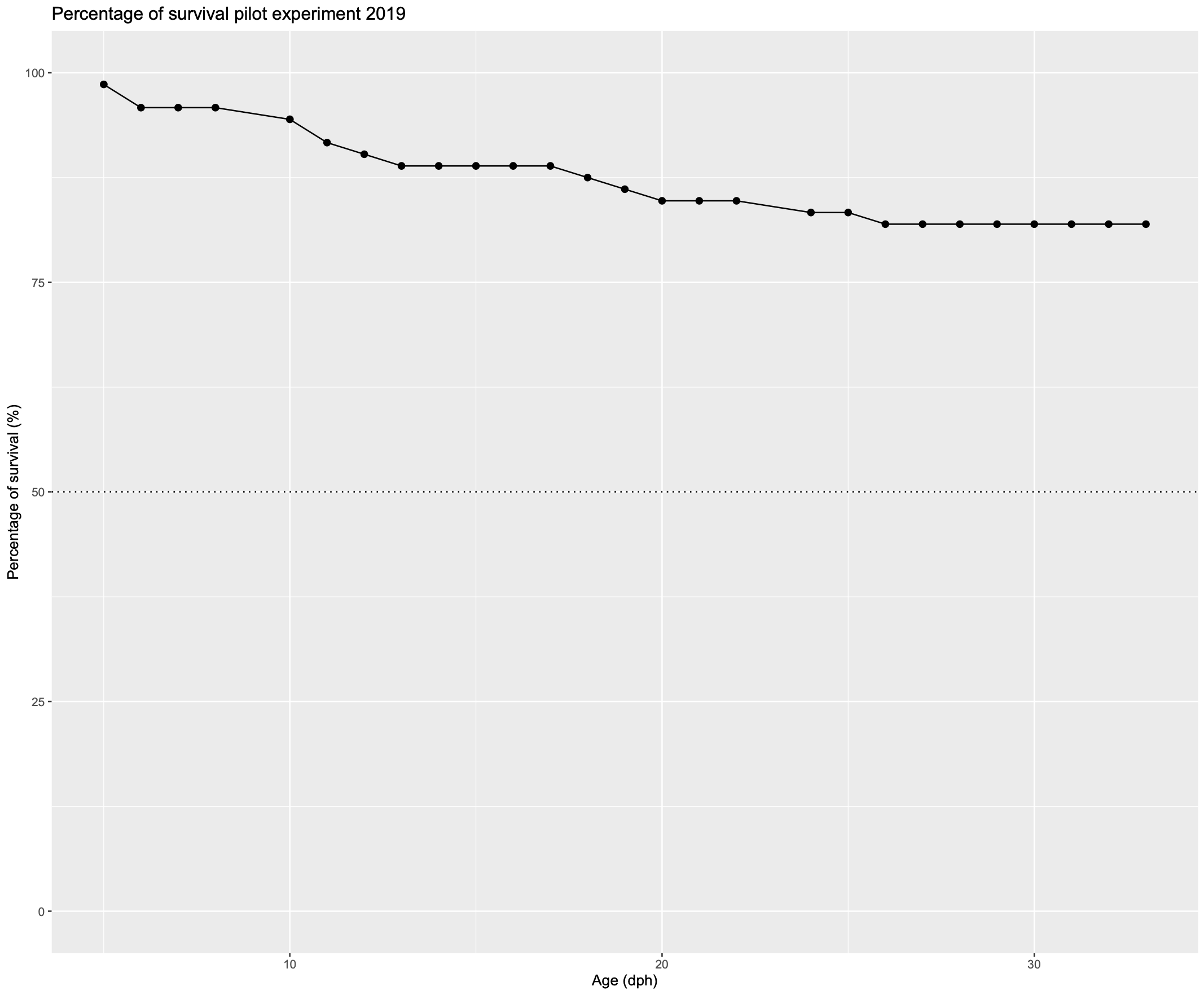


Figure S3. Percentage of survival of *Solea solea* larvae reared at optimal conditions (16ºC *ad libitum)* during the pilot experiment where we were testing the suitability of using 24-well microplates as housing system.

Figure S4. Comparison between experiments 1 and 2 (during the first 15 days) for the total percentage of survival of larvae reared at two temperatures and different feeding frequencies (grey) and proportion of larvae that fed six (blue), three (yellow) and twice a week (dark grey). During pilot 2, survival decreased rapidly, and after just three days, mortality was higher than 50% (defined as the lethal time for 50% of the individuals, LT_50_) in larvae reared at 20ºC, regardless of the food level (see LT_50_ values in days in Table 2). Generally, the LT_50_ in treatments at 16ºC doubled those obtained in the treatment resembling the RPC 8.5 scenario. Further, the first-week survival at 16ºC was 19% lower than the survival obtained during our pilot experiment (see Fig. S1).


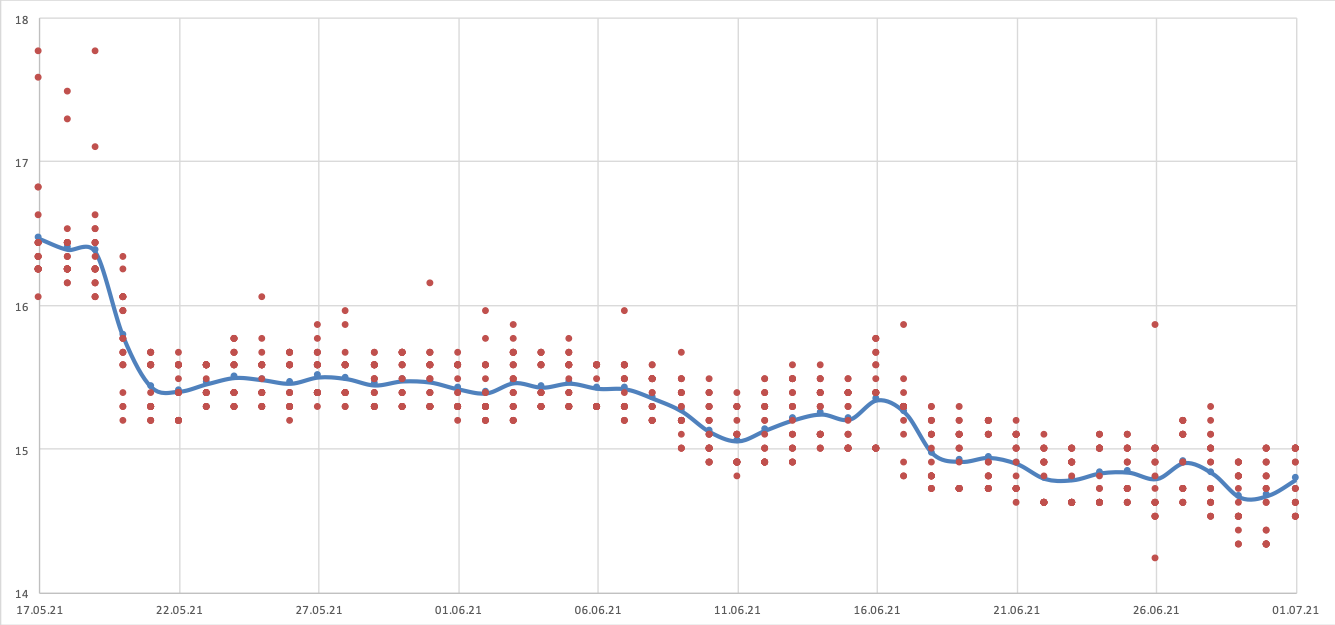


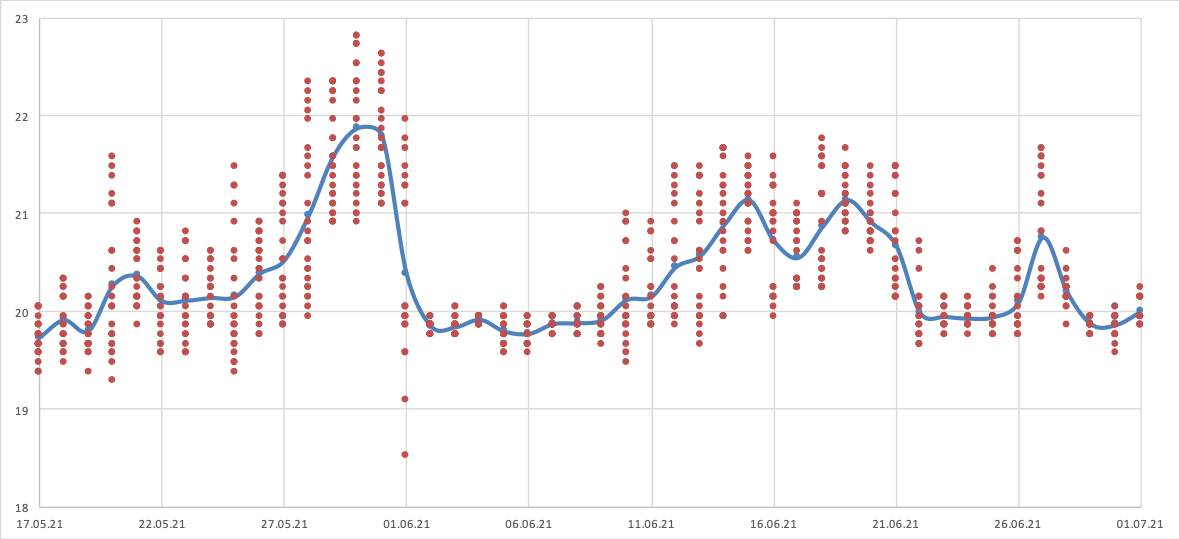


Figure S5. Daily temperature measurements in the rearing system for larvae reared at 16 (top) and 20ºC (bottom). Red points represent readings taken during the day and the blue line represents the daily average.
