# Supplementary material for: Temperature and feeding frequency impact the survival, growth, and metamorphosis success of Solea solea larvae
Source: PLoS One. 2023 Mar 23;18(3):e0281193. doi: 10.1371/journal.pone.0281193 (PMC10035848; doi:10.1371/journal.pone.0281193)
Supplement: Attachment — Submitted filename: Cover letter_Comments to reviewer.docx [file pone.0281193.s004.docx]

Dear Dr. Stampar,

Please find below the reply to all the reviewers’ comments specifying the modifications made in the revised manuscript as also the modifications made to meet Plos One format style.

Reviewer #1: The manuscript “Temperature and feeding frequency impact the survival, growth, and metamorphosis success of Solea solea larvae” by Sardi et al is a laboratory experiment with S. solea larvae targeting to endpoints such as survival, growth and metamorphosis.

The manuscript is well written and carefully justified in their procedure and findings. The authors also use a novel approach (proposed by other authors using 24 well plates) to be applied in ecotoxicology of larvae. They also propose a marine species as sentinel, other than the most frequently used fresh water model fish. The use of a more realistic natural species with high economic interest validates their effort as it could be applied in aquaculture and more particularly in larviculture. Nevertheless, their study also has some limitations such as the low quality of the stock egg production in some of their trials. They justify this as naturally occurring phenomena also in natural populations.

Their findings are not conclusive but their protocol and justification seem sound to me. Overall it is evident the large number of variables to be considered in laboratory experiments trying to predict biological consequences due to environmental variables fluctuations.

Thank you for your comments and your positive opinion on our work, which as you has mentioned targets to validate laboratory experiments for ecotoxicology, larviculture and in the general to contributing on using marine fish species as model species.

Other minor suggestions are:
Line 298. The sentence seems unfinished.

Indeed, it was incomplete, thank you for noticing.

Fig 2. The results for Temp of 20ºC are missing.

Apologies for this mistake, it seems to be an error while uploading the figure.

Fig 3 and 4 might be better replaced by a table informing on the characteristics of the slopes, correlations and significances under the different temperatures and feeding regimes in a more comparative way that the Figures reflect.

We appreciate the reviewer suggestion, but consider that for our objective —comparing the growth and weight among treatments— a figure is more appropriate than a table. Using figures allows for rapidly visualizing the differences, particularly because the axis limits are the same for all the figures, which gives a common ground for easily comparing the slopes.

Reviewer #2: Overall, this study was well designed and well written. I think it will contribute to this field.

Thank you for your time and for supporting our research.

However, some parts need minor revisions.
I will summarize some of them below,
The expressions in the abstract part but not suitable for this part should either be rearranged or moved to the relevant place.

We have placed the highlighted sentence in the first paragraph of the introduction as suggested.

There are some errors in the use and spelling of references in some parts. These need updating and editing.

Updated, thank you for noticing.

Adding photos of Solea solea larvae as figures can support the study visually.
Other recommendations are detailed in the attached file.

We have included a figure of a fully metamorphosed larva in the revised version.

1. Please ensure that your manuscript meets PLOS ONE's style requirements, including those for file naming. The PLOS ONE style templates can be found at

<https://journals.plos.org/plosone/s/file?id=wjVg/PLOSOne_formatting_sample_main_body.pdf> and

<https://journals.plos.org/plosone/s/file?id=ba62/PLOSOne_formatting_sample_title_authors_affiliations.pdf>

In addition to the comments from the reviewers, we have done the following formatting modifications in line with PlosOne guidelines.

1) Change the size of headings and subheadings to 18 and 16 pt respectively
2) Remove all points after the Fig citations
3) Bold the figure number and title of all figure legends. Include a figure title.
4) Move the figure legends immediately after the first paragraph citating the figure
5) Remove the figures from the manuscript file
6) Moved the tables from the end to immediately after the first citation
7) Swapped the order when citing figures or tables from the supplementary information document (i.e., S1 Fig instead of Fig S1)

2. In your Methods section, please provide additional information on the animal research and ensure you have included details on : (1) methods of sacrifice (2) methods of anesthesia and/or analgesia, and (3) efforts to alleviate suffering.

We have included details about the methods of sacrifice and concentration and chemical for anesthesia (line 239).

3. We note that you have indicated that data from this study are available upon request. PLOS only allows data to be available upon request if there are legal or ethical restrictions on sharing data publicly. For more information on unacceptable data access restrictions, please see [http://journals.plos.org/plosone/s/data-availability#loc-unacceptable-data-access-restrictions](http://journals.plos.org/plosone/s/data-availability#loc-unacceptable-data-access-restrictions).

In your revised cover letter, please address the following prompts:

a) If there are ethical or legal restrictions on sharing a de-identified data set, please explain them in detail (e.g., data contain potentially sensitive information, data are owned by a third-party organization, etc.) and who has imposed them (e.g., an ethics committee). Please also provide contact information for a data access committee, ethics committee, or other institutional body to which data requests may be sent.

This is not our case.

b) If there are no restrictions, please upload the minimal anonymized data set necessary to replicate your study findings as either Supporting Information files or to a stable, public repository and provide us with the relevant URLs, DOIs, or accession numbers. For a list of acceptable repositories, please see [http://journals.plos.org/plosone/s/data-availability#loc-recommended-repositories](http://journals.plos.org/plosone/s/data-availability#loc-recommended-repositories).

We have included the necessary data on supplementary information as an xls file.

We will update your Data Availability statement on your behalf to reflect the information you provide.

4. PLOS requires an ORCID iD for the corresponding author in Editorial Manager on papers submitted after December 6th, 2016. Please ensure that you have an ORCID iD and that it is validated in Editorial Manager. To do this, go to ‘Update my Information’ (in the upper left-hand corner of the main menu), and click on the Fetch/Validate link next to the ORCID field. This will take you to the ORCID site and allow you to create a new iD or authenticate a pre-existing iD in Editorial Manager. Please see the following video for instructions on linking an ORCID iD to your Editorial Manager account: <https://www.youtube.com/watch?v=_xcclfuvtxQ>

Done

5. Thank you for stating the following in the Acknowledgments Section of your manuscript:

“This study was funded by the IdEx Bordeaux International Post-doctorates program attributed to A. E. Sardi. The authors wish to thank Dr. Véronique Loizeau, Dr. Pierre Labadie and Dr. Florence Mounier for the fruitful conversations we held, which helped with the conceptualization of this work. “

We note that you have provided additional information within the Acknowledgements Section that is not currently declared in your Funding Statement. Please note that funding information should not appear in the Acknowledgments section or other areas of your manuscript. We will only publish funding information present in the Funding Statement section of the online submission form.

Please remove any funding-related text from the manuscript and let us know how you would like to update your Funding Statement. Currently, your Funding Statement reads as follows:

“A.S. was funded by the IdEx Bordeaux International Post-doctorates program. The funders had no role in study design, data collection and analysis, decision to publish, or preparation of the manuscript”

Please include your amended statements within your cover letter; we will change the online submission form on your behalf.

The funding statement is correct. I have removed the funding information from the acknowledgements section.

6. Please include your full ethics statement in the ‘Methods’ section of your manuscript file. In your statement, please include the full name of the IRB or ethics committee who approved or waived your study, as well as whether or not you obtained informed written or verbal consent. If consent was waived for your study, please include this information in your statement as well.

The Research and High Education French Ministry evaluated and approved all animal experimentations. The committee from the department of practical research activities licensed the project “Flatfish adaptation to temperature, feeding and pollution stress” led by Dr. Marie-Laure Bégout from Ifremer under the number reference APAFIS #38190-202208082103879.

This section is now included in the revised version of the manuscript.

7. Please include captions for your Supporting Information files at the end of your manuscript, and update any in-text citations to match accordingly. Please see our Supporting Information guidelines for more information: <http://journals.plos.org/plosone/s/supporting-information.>

The list of captions for the supporting tables and figures are now included at the end of the revised manuscript.

8. Please review your reference list to ensure that it is complete and correct. If you have cited papers that have been retracted, please include the rationale for doing so in the manuscript text, or remove these references and replace them with relevant current references. Any changes to the reference list should be mentioned in the rebuttal letter that accompanies your revised manuscript. If you need to cite a retracted article, indicate the article’s retracted status in the References list and also include a citation and full reference for the retraction notice.

9 We noticed you have some minor occurrence of overlapping text with the following previous publication, which needs to be addressed:

- <https://www.sciencedirect.com/science/article/abs/pii/S0304380020302945?via%3Dihub>

In your revision ensure you cite all your sources (including your own works), and quote or rephrase any duplicated text outside the methods section. Further consideration is dependent on these concerns being addressed.

Done
